# A Global, In-Market Evaluation of Toothbrushing Behaviour and Self-assessed Gingival Bleeding with Use of App Data from an Interactive Electric Toothbrush

**DOI:** 10.3290/j.ohpd.b2572911

**Published:** 2022-01-20

**Authors:** Susanne Thurnay, Ralf Adam, Michael Meyners

**Affiliations:** a Research Fellow, Procter & Gamble Service GmbH, German Innovation Center, Kronberg, Germany. Conceived the ideas and conducted the initial analysis, led the development of the manuscript, reviewed, edited and approved the final manuscript.; b Research Fellow, Procter & Gamble Service GmbH, German Innovation Center, Kronberg, Germany. Involved in interpretation of the data, reviewed, edited and approved the final manuscript.; c Principal Statistician, Procter & Gamble Service GmbH, German Innovation Center, Kronberg, Germany. Performed the detailed statistical analysis, led the development of the manuscript, reviewed, edited and approved the final manuscript.

**Keywords:** compliance, gum bleeding, in-market evaluation, interactive electric toothbrush

## Abstract

**Purpose:**

To determine if an interactive electric toothbrush and smartphone application (app) can reduce self-reported gingival bleeding and promote better brushing behaviour based on global, in-market usage data.

**Materials and Methods:**

Anonymised data were collected worldwide between July 2020 and January 2021 from users of interactive oscillating-rotating electric toothbrushes and app (Oral-B Genius, GeniusX and iO). Self-reported gingival bleeding and brushing behaviour data captured via the app were sent to Google Firebase and Google BigQuery to aid processing and analysis.

**Results:**

Data from 16.7 million brushing sessions were analysed. 439,481 new users responded at least once to the app question: ‘Do you have gum bleeding?’ Of users answering the question over their first two weeks of app use (153,201), the proportion reporting bleeding decreased statistically significantly from week 1 to 2 (28.8% to 17.1%, p < 0.0001). Of users answering the question over each of the first five weeks (43,060) a further statistically significant decrease in those reporting bleeding was seen in each consecutive week, with the week-5 rate being 12.7% (p < 0.0001 vs any previous week). Decreases in duration of excessive pressure (i.e. > 2.5 N – 3.0 N depending on the handle) decreased the proportion of self-reported gingival bleeding (p < 0.0001). Users brushed longer and with less overpressure (p < 0.0001) with vs without live feedback from the app, and showed 94.4% average coverage with live feedback.

**Conclusion:**

The interactive oscillating-rotating electric toothbrushes and app, particularly with live feedback, promote good brushing behaviour. Self-reported gingival bleeding occurred less frequently the longer the system was used.

Gingival bleeding while brushing is relatively easily observed by consumers and is a common early signal of gingivitis, which is typically caused by inflammatory reactions due to accumulation of dental plaque.^27,39,40^ It is described in the literature that self-assessment of gingival bleeding while brushing is a clear indicator for bleeding-on-probing,^16,41^ and research among youth has shown that self-reported gingival bleeding may be useful for monitoring and promoting gingival health.^25,26,38^ Gingivitis is known to be largely preventable and can be reversed, but does require a thorough daily oral hygiene routine with the effective mechanical removal of dental plaque.^6,7,32^ Relative to a manual toothbrush, the electric (i.e. power) toothbrush is well recognised for its potential to offer the user a more effective means of achieving good plaque control.^43^ Among the different models of marketed electric toothbrushes, those with oscillating-rotating action have been shown to provide advantages for plaque control and improved gingival health.^8,9,19^

Traditional educational attempts to establish and promote effective brushing behaviours have demonstrated limited outcomes.^10,11^ Continuous on-the-spot education and training, as well as person-centered, individualised approaches where the patient takes an active role in behaviour change,^21^ might achieve more than just oral hygiene instruction in professional settings every few of months. This idea has been pursued by other researchers who developed interactive devices for at-home oral hygiene and has proven to take time and effort.^18,35^ Improving brushing behaviour and raising awareness that gingival bleeding on brushing is a potentially serious oral hygiene concern have been important considerations in the development of a smartphone application (app; Oral-B version 8.x) for use with the most recent models in the oscillating-rotating series of Oral-B electric toothbrushes: Genius (D701.6 [subsequently G]), GeniusX (D706; GX) and iO (M7, M8, M9; iO). Following the initial brushing session, consumers using the app are asked if they have ‘gum bleeding’. This question is intended to draw attention to the condition. The app allows users to track gingival bleeding incidents with repeat questions and, if bleeding persists, users are advised to visit their dental health professional.

The technology also includes timers to guide and encourage users to brush for a sufficient duration, pressure sensors that warn users if more brushing pressure than recommended is being used, and a position detection function that identifies areas that are being neglected during brushing and enables the user to brush more evenly throughout the mouth. The advantage, therefore, for the individual consumer using this technology is that it can provide real-time (live) feedback with brushing guidance to help the user achieve a highly effective, at least two-minute, twice-daily brushing habit which is recommended for maintenance of good gingival health.^4,42^

This evaluation of gingival bleeding and brushing behaviour data, which were collected anonymously from the global user population, aims primarily to determine whether the interactive technology of these oscillating-rotating electric toothbrushes can be used to reduce gingival bleeding incidents as reported by consumers and help to promote better brushing behaviour (e.g. brushing for 2 min, brushing entire dentition, using appropriate force).

## Materials and Methods

### Subjects and Materials

**Fig 1:**
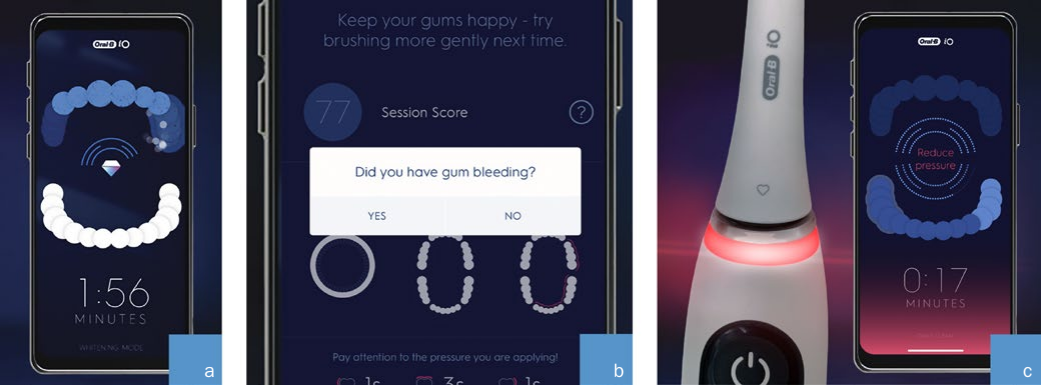
Brushing behavior features: (a) position detection on app screen; (b) gum bleeding question on app screen; (c) visual pressure sensor illuminates red on LED SmartRing and app screen if too much pressure is applied.

Global consumer usage data collected anonymously from users of three oscillating-rotating toothbrushes (G, GX, iO) and their associated app on a smartphone were used for this evaluation. Given that no additional survey or demographic data were available, and as data were anonymous, no exclusion criteria could be applied. The only inclusion criterion was that the app was used at least once during the respective period and had not been used by the same subject before (at least with the same handle, which was used as the identifier, see below). This implies that subjects may have used the toothbrush before, and possibly even for a significant period, but without the app. Brushes G and GX share the same drive design and use interchangeable brush heads. Like G and GX, iO uses oscillating-rotating technology, which is clinically proven to be effective in removing dental plaque and improving gum health,^19^ but has a linear magnetic drive directing energy to toothbrush bristles to optimize plaque removal.^1^ In addition, all three brushes provide real-time visual feedback when too much force is used by illuminating, in red, the neck of the brush and the app screen (see Table 1 and Fig 1). The iO also provides visual feedback via a green light when the ideal amount of pressure is applied and via white light (or an alternate color selected by user) when too little pressure is used.

**Table 1:** Description of key app features

App features	Description	
Pressure feedback	Pressure control sensors are activated when brushing is too hard (i.e. greater than target pressure of about 2.5–3.0 N, depending on handle). Visual feedback is provided in real time by illuminating, in red, the neck of the brush (the LED SmartRing) and the app screen. The technology also works automatically to slow the brush and, for G and GX, to stop its pulsating action when there is overpressure. Duration of overpressure is tracked.	
Gum bleeding tracking	Yes/no question asked after initial brushing and repeated based on response. Consumer can choose different repeat frequency if desired.	
Feedback type (choice of 1 per brushing session for G; for GX and iO, position detection is always on in live sessions)	Timer only On-screen pacer to guide user to spend the same amount of brushing time in each of either four or six (depending on user’s choice) sections of the mouth, for a total brushing time of 2 min. Actual position not tracked.	Position detection App tracks position of the brush within the mouth in real-time during brushing and shows what areas still need to be brushed to encourage better coverage and evenness.

No demographic or other personally identifiable information was collected. The global privacy policy of the manufacturer (Procter & Gamble; Cincinnati, OH, USA) was followed, and additional local privacy requirements were adhered to as appropriate. Media Access Control (MAC) addresses of the handles were used to track users over time. These MAC addresses are not read out during manufacturing or product distribution, thus no link to the user of the handle is possible. Google infers location information (country) from the IP address of the phone used, but the IP addresses themselves are not part of the data available for evaluation.

These electric toothbrushes use Bluetooth connectivity to communicate brushing activity data to the app and give brushing feedback to the user aimed at achieving better brushing behaviour. The app has been designed with several features, the key ones being listed in Table 1 and shown in Fig 1.

In Brush G, the position detection feature functions are based on facial recognition and sensors in the toothbrush handle. Users place their phone in a holder at eye level and then brush in front of the phone. An algorithm trained on thousands of brushing sessions analyses the data from the sensors and the phone camera and calculates the position of the brush within the mouth.

Brushes GX and iO have additional sensors in the handle so that facial recognition is not needed. Users still need to have their phone at hand, but they do not need to have it at eye level, and they can move around while brushing. An algorithm trained again on thousands of brushing sessions analyses the data from the sensors to calculate the position of the brush in the mouth.

When the position detection feature is used, the app gives live feedback to the user on the amount of time spent brushing, and whether brushing is too hard, for each of five (G) or six (GX, iO) zones tracked within the mouth: left maxillary (upper), left mandibular (lower), right maxillary, right mandibular, and (maxillary and mandibular) front (combined as one zone for G). At the start of brushing, the zones are blue in color and become ever lighter while brushing until they become white. The aim of the position detection technology is to guide the user while brushing to cover the mouth evenly without overpressure and not to miss any zones. For one of the iO versions (M9) only, additional information on lingual vs occlusal vs buccal coverage is provided to the user, but that data is not considered in this analysis.

The app tracks gingival bleeding incidents of the user by asking ‘Did you have gum bleeding?’ (‘yes’ or ‘no’) and storing the result. Those users who reply ‘no’ are asked the question only once a week thereafter, to avoid disturbing them with too many repeat questions. Those users who reply ‘yes’ are asked the question after every brushing session, until they have responded ‘no’ 4 times consecutively, after which they are asked once a week. Users also have the option to manually set the reminder frequency to daily, weekly, monthly, or off.

The study protocol has been submitted for IRB evaluation, and in the Board’s opinion, the work presented is not subject to FDA regulation within the scope of 21 CFR and does not meet the definition of human subjects research at 45 CFR 46.102, and therefore no IRB supervision was required (Advarra IRB, letter from April 6, 2021). The IRB evaluation was limited to the use of the already existing data and data collected in accordance with national regulations. IRB evaluation was performed prior to analysis for this research, but after (ongoing) data collection.

### Statistical Methods

Brushing data were recorded by the Oral-B app. It is possible that, at times, different subjects from the same household used the same toothbrush handle. If done consistently (e.g. a couple using the same handle but different brush heads), results related to such a handle would be more variable yet still give the same trend as if two handles were used. If this occurs only occasionally, it may dilute overall results, but given the large dataset and this situation being expected to be rare, we do not expect noteworthy implications on interpretation. The data used for this research were gathered from 1 July 2020 to 27 January 2021 (chosen for homogeneity of data which is impacted by release dates of various app versions and toothbrushes). The users’ app data were sent to Google Firebase, a web analytics platform for tracking and viewing data, and to Google BigQuery, a computing tool that allowed the data to be manipulated for analysis. All statistical analyses were performed using R 3.6.3.^31^ All statistical tests were two-sided with a significance level of 0.05; confidence intervals are accordingly at the 95% level. Bonferroni-Holm correction for multiplicity was used within each analysis; however, it was at times restricted to subsets with reasonably robust sample sizes.

Results for the ‘gum bleeding’ question were summarised, by week and also by country, as the number of users who answered the question and the proportion of those users who reported gingival bleeding at least once during that week (‘yes’), vs those who did not report any gingival bleeding during that week (‘no’). Comparisons between different weeks were confined to users answering the question on gingival bleeding at least once in each of the respective weeks. Differences in proportions of users reporting gingival bleeding at least once during the respective week were compared using McNemar’s test (employing an exact binomial test rather than a chi-squared approximation). The Bonferroni-Holm correction for multiplicity was restricted to those countries with more than 200 users in our data base for this analysis to avoid dilution of results due to countries with low sample sizes.

Gingival bleeding rates were compared between brush G and GX (same technology) on the one hand and iO on the other. As the rate at which consumers are asked by the app to report potential gingival bleeding depends on their previous responses, it might have biased the comparison due to different recurrences of the question. Instead, we used only the data from the first time a consumer was asked during any week of usage. Unlike the previous analysis based only on consumers providing data for all weeks under consideration, here, all consumers providing any data for the respective week were included. A chi-squared test for the comparison of two independent proportions with continuity correction was used for each week, and p-values were corrected across weeks using the Bonferroni-Holm procedure.

Based on brushing sessions with an answer to the binary (i.e. ‘yes’/’no’) gingival bleeding question, the relationship between the duration of overpressure and the occurrence of bleeding was analysed using logistic regression. To avoid the results being primarily driven by users with very extended overpressure durations, the analysis was repeated on data restricted to brushing sessions with less than or equal to 5 s (1 s) of total overpressure duration.

Brushing coverage was calculated according to the zones of the dentition considered (5 for G, 6 for GX and iO). The overall brushing target time was 120 s, but the target time for a zone depended on the number of surfaces for that zone. Coverage was defined as the percentage of the target time for that zone that the user actually brushed that zone, with a maximum of 100% even if the actual brushing time exceeded the target time. Using actual and target times for each zone, the percentage brushing coverage for each session was calculated as the arithmetic average across the coverages determined for the 5 (G) and 6 (GX, iO) zones.

Coverage was defined as ‘complete’ (yes/no) for any session if all zones were brushed for at least the target time during that session.

Brushing coverage and complete coverage were calculated using data collected when using the position detection feature. Total brush duration data and overpressure duration were also recorded when using the regular timer on the app and for offline sessions (i.e. without concurrent use of the app). The data for brushing duration and overpressure duration (raw values as well as percentage of total duration) were compared using a mixed-effect model with brushing type (position detection, timer only or offline) and handle series (G, GX or iO) as fixed effects and subject as random effect, using REML estimation.

## Results

During the data collection period, the app provided brush usage data for about 16.7 million brushing sessions, 7.6 million of which used the position detection feature. A total of 439,481 new users answered the app question ‘Do you have gum bleeding?’ at least once and, in total, they provided 3,906,933 answers to the question for up to 30 consecutive weeks.

Table 2 shows the number of users who answered the gingival bleeding question at least once in each of the first two weeks they used the app, i.e. weeks 1 and 2. Numbers are shown both overall and for the 35 countries with respective data for at least 200 users. Users who answered the question in week 1 only or week 2 only were excluded. Also shown are the numbers and percentages of those users who reported gingival bleeding at least once a week. There was a statistically significant decrease in users reporting gingival bleeding within a week of usage of the toothbrush when the data were analysed for all users (decrease from 28.8% with 95% confidence interval [28.6%, 29.1%] to 17.1% [16.9%, 17.3%]), as well as when the data were analysed separately for each individual country (p < 0.0001 throughout after multiplicity correction across all 36 countries, including ‘all’, with at least 200 users, except for Hungary with a corrected p-value of 0.0013).

**Table 2:** Users who answered the ‘gum bleeding’ question at least once in each of weeks 1 and 2 of app use and comparison of proportions of users reporting gum bleeding at least once within the respective week

Country	Users who answered question	Users reporting gum bleeding			
		Week 1		Week 2	
	n	n	%	n	%
All	153,201	44,180	28.8	26,219	17.1
United States	55,948	16,165	28.9	8,811	15.7
United Kingdom	20,278	6,298	31.1	4,034	19.9
Germany	20,067	4,323	21.5	2,658	13.2
Italy	7,239	2,068	28.6	1,259	17.4
Canada	5,440	1,548	28.5	912	16.8
Netherlands	3,995	885	22.2	515	12.9
Japan	3,450	903	26.2	579	16.8
Spain	3,169	1,014	32.0	662	20.9
France	2,979	792	26.6	524	17.6
China	2,890	1,316	45.5	845	29.2
Sweden	2,595	743	28.6	491	18.9
Belgium	2,390	803	33.6	516	21.6
Australia	2,262	706	31.2	416	18.4
Norway	2,228	657	29.5	411	18.4
Austria	1,874	451	24.1	290	15.5
Switzerland	1,578	387	24.5	254	16.1
Denmark	1,444	400	27.7	270	18.7
Czech Republic	1,365	476	34.9	259	19.0
Finland	1,352	355	26.3	257	19.0
Russia	1,119	433	38.7	229	20.5
Poland	801	216	27.0	126	15.7
Turkey	762	314	41.2	171	22.4
Romania	681	234	34.4	141	20.7
Taiwan	602	358	59.5	214	35.5
Ireland	573	190	33.2	106	18.5
Israel	569	221	38.8	137	24.1
Hong Kong	567	261	46.0	151	26.6
Hungary	449	118	26.3	86	19.2
Singapore	419	134	32.0	59	14.1
South Korea	339	96	28.3	47	13.9
Portugal	289	107	37.0	50	17.3
New Zealand	268	84	31.3	46	17.2
Greece	264	107	40.5	64	24.2
Saudi Arabia	257	115	44.7	71	27.6
United Arab Emirates	236	82	34.7	40	16.9

p-values from McNemar’s test after Bonferroni-Holm correction across all 36 countries shown (including ‘all’) were all ≤ 0.001.

Of the 439,481 users who answered the gingival bleeding question, a total of 43,060 answered the question at least once per week for the first five consecutive weeks. At week 1, 28.1% of these users reported gum bleeding at least once; at week 2, the percentage had decreased to 18.7%. This downward trend in the number of users who reported gingival bleeding continued with a statistically significant decrease (p <0.0001) in each consecutive week with reported gingival bleeding for weeks 3, 4 and 5 of 16.3%, 14.9% and 12.7%, respectively. The same trend was observed by country for those countries with at least 200 users, with p-values for the change from week 2 to week 5 after multiplicity correction across the 26 countries (including ‘all’) being <0.001 for 14 countries and > 0.1 only for those 5 countries with the lowest absolute average count across weeks 2 and 5.

**Fig 2:**
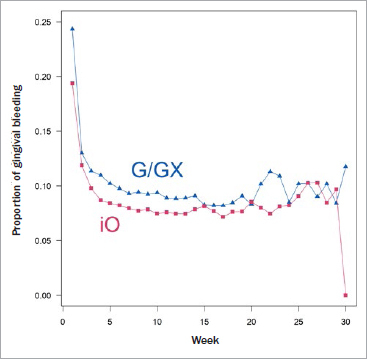
Proportion of observed gingival bleeding for iO and G/GX at first time asked during a week of usage across weeks. p-values for the comparison between proportions per week after multiplicity correction are < 0.001 for the first 10 weeks and < 0.05 through week 13.

Figure 2 shows the proportion of self-reported gingival bleeding rates between G/GX and iO by week, based on all available observations regarding the first time a consumer is asked during that week. We consistently observe lower rates for iO compared to G/GX up to week 19; up to week 13, these differences were statistically significant based on the chi-squared test and after correcting for multiplicity according to the approach of Bonferroni-Holm. For later weeks, the results became less stable; this can be attributed to sample sizes which fell (far) below ca. 4000 for G/GX and below 2000 for iO as of week 20, and dropped to 170 and 8 in week 30, respectively. These sample sizes are insufficient to reliably estimate the respective rates or even discriminate between them based on the observed difference.

**Fig 3:**
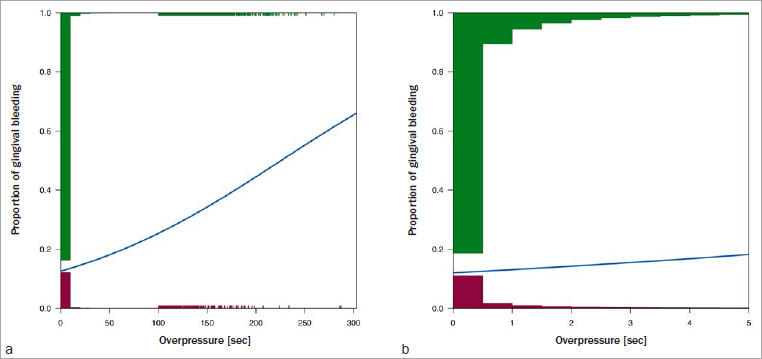
Estimated curves and histograms illustrating the distribution of responses with (red) and without (green) gum bleeding and probability of gum bleeding in relation to overpressure duration. a: all responses, b: responses with duration of overpressure ≤ 5 s. Note that in a, the histogram covers only the first 100 s; after that, individual events are displayed by small ticks.

Figure 3 shows the logistic fit of duration of overpressure and the probability of bleeding for those sessions with responses to the gingival bleeding question. Histograms illustrate the distribution of responses, with gingival bleeding shown upwards in red and no gingival bleeding shown downwards in green. Figure 3a (3,906,931 responses) shows that longer durations of overpressure, although rare, had a higher probability of being brushing sessions with bleeding (p < 0.0001). The reduced scales used to illustrate the duration of overpressure in Fig 3b (3,732,269 observations) show that increases in duration of even short bursts of overpressure (up to 5 s) were statistically significantly (p < 0.0001) associated with increased probability of gingival bleeding. The same trend was observed when confining the analysis to sessions in which the overall overpressure duration did not exceed 1 s (details not shown). Table 3 shows the details from these analyses, also highlighting that while the intercept is about the same across the analyses, the slope becomes much steeper when observations with long overpressure are omitted from the analysis (note that this is not obvious from Fig 2, as the range of the x-axis is different between A and B), suggesting that the effect is already present with relatively short overpressure duration, and its size to some extent diluted when also taking long durations into account.

**Table 3:** Results from logistic regression of gum bleeding depending on overpressure duration

Data	Intercept			Slope		
	Estimate	SE	95% confidence interval	Estimate	SE	95% confidence interval
All	-1.940	0.002	-1.943 -1.937	0.0086	0.0002	0.0080 0.0091
Overpressure up to 5 s only	-1.990	0.002	-1.993 -1.987	0.0974	0.0016	0.0942 0.1006

Analyses rely on all data, and on data with ≤ 5 s overpressure, respectively.

Table 4 shows the results for the analysis of coverage, brushing duration and overpressure duration data for brushing sessions using the position detection, timer only and offline sessions (note that coverage data is only available in position detection mode). Sessions using position detection showed a very good average coverage of 94.4%, with almost 80% of sessions with complete coverage. Brushing duration was found to be statistically significantly longer by about 30-40 s (i.e. 25%-30% increase) when using the position detection feature than when using either the timer only or when brushing without using the app. Simultaneously, and despite the longer brushing duration, the duration of overpressure was statistically significantly lower with position detection. The occurrence of overpressure was the largest by far when not using the app at all while brushing, which is more easily seen when evaluating overpressure duration as percentage of total duration.

**Table 4:** Comparison of brushing measures obtained for the different technological features: position detection, timer only and offline sessions

Brushing measure	Number of brushing sessions	LS Means^§^		
		Position detection	Timer only	Offline session (app not used)
Coverage (%)	7,568,598^†^	94.4		
Sessions with complete coverage (%)	7,568,598^†^	79.6		
Duration (s)^§^	16,681,216^‡^	162.6	122.7	133.8
Overpressure (s)^§^	16,681,213^‡^	1.55	1.64	2.35
Overpressure (% of total duration)^§^	16,681,213^‡^	1.06	1.35	2.04

^†^Total sum of sessions with position detection. ^‡^Total sum of sessions with non-missing data: guided + timer-only + offline. ^§^For (complete) coverage, values are just the plain mean values. For duration and overpressure, these are the LS means from the mixed model. All two-sided p-values for the pairwise comparisons between these means are < 0.0001 after Bonferroni-Holm correction for multiplicity across all 9 pairwise comparisons (3 for duration and both representations of overpressure each).

## Discussion

Published research has shown improvements in brushing technique and increased brushing time among adolescent and orthodontic patients using earlier models of interactive oscillating-rotating electric toothbrushes with an app.^12,13^ With the development of the oscillating-rotating electric toothbrush and its accompanying smartphone app, the brush manufacturer for the first time interacted with consumers about self-reported gingival bleeding, tracking and recording their brushing data for them, and giving live feedback in the form of brushing guidance based on a generally available combination of toothbrush and app for in-home use without any dedicated intervention. An important benefit is that using an app not only allows the provision of proper brushing instruction (e.g. time) but also tracks behavioural aspects, which highlight the effectiveness of the brush/app combination. Upon brushing without the app, only features such as the pressure sensor could be used; in that case, it is not possible to assess adherence to and effects of recommendations. The worldwide availability of this brush technology presented a unique opportunity to raise awareness among consumers, across a wide population, about gingival bleeding on brushing as an oral hygiene concern that requires attention, thereby contributing to efforts preventing periodontal diseases.^2,33^ Unprecedented volumes of anonymised data from real consumer usage around the globe were available, and they enabled us to investigate the impact of using brushes G, GX, or iO and app on at-home brushing behaviour and self-reported gingival bleeding.

The lower rate of self-reported gingival bleeding observed with iO relative to the Genius models at least over the first 12 weeks is likely due to the combination of clinical efficacy and features to improve brushing behaviour, including the additional feature of iO to also highlight underpressure. Gingival bleeding is typically a function of inflammation and/or mechanical trauma, and areas of disease are often more susceptible to bleeding from mechanical force. This analysis showed that increases in overpressure and hence likelihood of mechanical trauma were associated with increased probability of gingival bleeding. The iO is the only brush among the three to provide positive feedback when correct pressure is used for most effective cleaning and thereby prevention of plaque and gingival bleeding, and it also offers the most advanced technology with the linear magnetic drive among this family of oscillating-rotating toothbrushes.^1,17^

The current in-market evaluation showed that with on-going use, consumers achieve better brushing behaviour and better outcomes (i.e. less self-reported gingival bleeding) with these oscillating-rotating electric toothbrushes and app. Reductions in self-reported gingival bleeding were seen across more than 30 countries representing 4 continents, indicating the technology is effective and easy-to-use across cultures. Brushing behaviour improvements were strongest when using live guidance. We postulate this is because live guidance directs behaviour change during usage, at a time when consumers have the greatest ability to act upon it. Research shows that feedback via digital technology is an effective approach to disrupt habits, but there is less clarity around how long the habit change is sustained.^20^ Live guidance via an app seems to offer a way to consistently provide feedback, thereby supporting longer-term habit changes (i.e. at least as long as the app is used).

A limitation of this evaluation is that it is impossible to know whether an individual user was brushing with an electric brush before starting to use brush G, GX or iO and app or whether they switched from a manual. The population is likely a mix of consumers who are experiencing the gingival health benefit of their first oscillating-rotating electric toothbrush as well as the brushing behaviour benefit of the app and consumers who come from an oscillating-rotating electric toothbrush and are experiencing only the brushing behaviour benefit of the app. While we have shown that the live guidance available only via the app does lead to brushing behaviour improvements, the gingival bleeding reduction is likely driven by a combination of both effects. The present findings align with those on oral health benefits of electric toothbrush use,^8,9,17,19,43^ as well as observations from long-term observational studies in which electric toothbrush users showed less gingival disease than manual toothbrush users.^22-24,30^

Another potential limitation of this work is that the sample may be biased with regard to demographic variables such as age and income. Younger consumers and those with higher socioeconomic status might be over-represented in the sample. For example, younger people have been reported to track their health via an app or fitness trackers more often than the elderly.^5,35-37^ Whether and to what extent such a bias also occurs for a combination of a premium toothbrush with an app is unclear and cannot be investigated based on the present evaluation, as no demographic data were collected. Generalisation to an entire population is hence difficult, in particular as other demographic groups might interact with an app differently, if at all. Nevertheless, the results can be considered as reasonably generalisable for the general part of the population that is able and willing to purchase and interact with a premium electric toothbrush and use it in combination with an app in the intended way. As in any other research, generalisation is only possible to the population from which the data were sampled. In this real-world-evidence research, we do not have control over demographic or other additional details; it is, however, reasonable to assume that the sampling and hence the results largely hold for the target audience, i.e. people able and willing to acquire a premium product for toothbrushing and use it in combination with an app.

The data presented complement results of clinical trials conducted on oscillating-rotating electric toothbrushes that have consistently revealed advantages for plaque removal and gingival health over manual and other electric toothbrush models.^8,9,17,19,43^ The present evaluation, with data from the regular oral hygiene routines of users, therefore served to link experimental findings from clinical trials to the real world of consumer bathrooms. One obvious advantage of this approach is that it was carried out as users performed their daily brushing regimen without the attention of professionals, and thus avoiding alterations in consumer behaviour that can arise when users are being formally studied, a phenomenon known as the Hawthorne effect.^15,29^ A further strength of the present evaluation was that it assessed gingival bleeding as reported by consumers themselves (using the consumer term ‘gum bleeding’), which made this an evaluation with an efficacy endpoint that was both a tangible outcome and patient-centered. At the same time, this is also a limitation, as the self-reported incidents of gingival bleeding cannot be objectively confirmed in the present evaluation. Nevertheless, a positive correlation between self-reported bleeding on brushing and clinical bleeding on probing has been previously found.^16,41^

The interactive technology not only allows large populations of consumers to be questioned remotely and anonymously about brushing outcomes such as gingival bleeding and/or behaviour (e.g. whether they flossed) while they carry out their daily brushing regimen, but can also be used to give individual consumers focused live feedback to improve their oral care habits. This feedback is based on data collected on aspects of brushing behaviour that would otherwise prove difficult to obtain and analyse.

Although the users in the present evaluation were a relatively narrow subset of consumers, i.e. those who could afford to purchase a premium product and who were sufficiently motivated to interact and comply with an app while brushing, the findings have wide applicability. Gingivitis is a common periodontal disease and well recognised as a major oral healthcare problem, affecting up to 80% of populations worldwide.^3,14,28^ Identifying ways of reducing the global burden of this disease is an ongoing challenge to oral health care professionals. Findings from this evaluation serve to highlight the potential benefits of heightened consumer awareness for the signs of poor gingival health and the need for self-motivation in improving brushing behaviour. Crucially, the evaluation identified measures of brushing behaviour that were improved with use of the interactive electric toothbrush and app, including excessive pressure duration and brushing coverage. Studies of systematics in toothbrushing behaviour show that the latter two are critical components of effective toothbrushing.^34^ Importantly, the gingival health benefit seen in this evaluation was both patient-centered (i.e. observable) and, although not objectively confirmed through clinical evaluation, seems meaningful to consumers.

## Conclusion

In this analysis of over 16 million real-world, anonymised brushing sessions collected worldwide, the use of an app in combination with an interactive oscillating-rotating electric toothbrush was found to be associated with decreased gingival bleeding and improved brushing behaviour, particularly when used with live feedback. These data supplement findings from clinical trials which also demonstrate improvements in gingival health and brushing habits with use of the toothbrush and app technology. When incorporated in patients’ daily oral hygiene routine, these interactive oscillating-rotating toothbrushes provide an important tool to help patients achieve and maintain gingival health and positive oral hygiene behaviors.
